# DNA from KI, WU and Merkel Cell Polyomaviruses Is Not Detected in Childhood Central Nervous System Tumours or Neuroblastomas

**DOI:** 10.1371/journal.pone.0008239

**Published:** 2009-12-11

**Authors:** Géraldine Giraud, Torbjörn Ramqvist, Diana V. Pastrana, Vincent Pavot, Cecilia Lindau, Per Kogner, Abiel Orrego, Christopher B. Buck, Tobias Allander, Stefan Holm, Bengt Gustavsson, Tina Dalianis

**Affiliations:** 1 Department of Oncology-Pathology, Karolinska Institutet, Stockholm, Sweden; 2 Laboratory of Cellular Oncology, Center for Cancer Research, National Cancer Institute, Bethesda, Maryland, United States of America; 3 Department of Microbiology, Tumor and Cell Biology, Karolinska Institutet, Stockholm, Sweden; 4 Department of Women and Child Health, Karolinska Institutet, Stockholm, Sweden; 5 Department of Neurosciences, Karolinska Institutet, Stockholm, Sweden; 6 Department of Virology, Swedish Institute for Infectious Disease Control, Solna, Sweden; The University of Chicago, United States of America

## Abstract

**Background:**

BK and JC polyomaviruses (BKV and JCV) are potentially oncogenic and have in the past inconclusively been associated with tumours of the central nervous system (CNS), while BKV has been hinted, but not confirmed to be associated with neuroblastomas. Recently three new polyomaviruses (KIPyV, WUPyV and MCPyV) were identified in humans. So far KIPyV and WUPyV have not been associated to human diseases, while MCPyV was discovered in Merkel Cell carcinomas and may have neuroepithelial cell tropism. However, all three viruses can be potentially oncogenic and this compelled us to investigate for their presence in childhood CNS and neuroblastomas.

**Methodology:**

The presence of KI, WU and MCPyV DNA was analysed, by a joint WU and KI specific PCR (covering part of VP1) and by a MCPyV specific regular and real time quantitative PCR (covering part of Large T) in 25 CNS tumour biopsies and 31 neuroblastoma biopsies from the Karolinska University Hospital, Sweden. None of the three new human polyomaviruses were found to be associated with any of the tumours, despite the presence of PCR amplifiable DNA assayed by a S14 housekeeping gene PCR.

**Conclusion:**

In this pilot study, the presence of MCPyV, KI and WU was not observed in childhood CNS tumours and neuroblastomas. Nonetheless, we suggest that additional data are warranted in tumours of the central and peripheral nervous systems and we do not exclude that other still not yet detected polyomaviruses could be present in these tumours.

## Introduction

Polyomaviruses are DNA tumour viruses that were first described in humans with the simultaneous discovery of JC virus (JCV) and BK virus (BKV) in 1971 [1], [2]. JCV has a unique tropism for replication in glial cells and its replication in humans can cause progressive multifocal leucoencephalopathy (PML), a fatal demyelinating disease of the central nervous system (CNS) in immunosuppressed patients [2], [3]. During three decades of research, JCV has been shown to transform cells in culture, particularly cells of glial origin and to have a highly oncogenic potential in laboratory animals (reviewed in [4]). Moreover, in humans, JCV has been associated with CNS tumours [5], [6], but so far, the data are inconclusive to pinpoint such an association [7], [8], [9], [10], [11]. BKV is associated with nephropathy and hemorrhagic cystitis in renal and allogeneic haematopoietic stem cell transplant recipients, respectively (reviewed in [3]). In newborn rodents, BKV is also highly oncogenic, and although it can be found in experimental tumours of the CNS its association to the nervous system is assumed to be weaker than that of JCV [3], [12], [13]. Moreover, a possible role for BKV in the aetiology of embryonal neuroblastomas of the sympathetic nervous system has been suggested [14] but also disputed [15] and similarly studies on BKV in human brain tumours present conflicting and inconclusive results (reviewed in [16]).

Recently, three new polyomaviruses, KI, WU and MC polyomaviruses [17], [18], [19] have been identified in humans. These three viruses are, with regard to protein sequences, rather different from JCV and BKV, with KI and WU being most closely related, and MCPyV diverging from all earlier human polyomaviruses [20].

To date, KI and WU polyomaviruses (KIPyV and WUPyV) have not been associated to human diseases. Although many reports have confirmed their discovery in nasopharyngeal aspirates from patients suffering from acute respiratory diseases, so far the data do not suggest that KIPyV and WUPyV are aetiological agents for acute respiratory diseases [3], [21], [22], [23] and there is still limited information regarding the tropism of KIPyV and WUPyV [20]. Nevertheless, as members of the polyomavirus family, KIPyV and WUPyV have all the qualifications to be cofactors in the induction and/or progression of human tumours.

With the discovery of MCPyV in 2008, for the first time a strong association between a human cancer and a polyomavirus was demonstrated and later confirmed by several groups (reviewed in [3]). The presence of MCPyV in Merkel cell carcinomas (MCC), its integration [19], [24] and its clonal mutation in the C-terminal region of the Large T antigen [25], merit further investigation both on the epidemiological and *in vitro* level in order to conclude in a direct oncogenic role of this polyomavirus according to the criteria of Harald zur Hauzen (detailed in [16]). There is limited information regarding the tropism of MCPyV, which can also be found in nasopharyngeal aspirates [26], [27], [28], [29], nonetheless the discovery in Merkel cell carcinomas indicates a tropism for neuroepithelial cells. Several studies have been conducted to investigate for the presence of MCPyV in other human tumours. So far MCC is the only tumour type where MCPyV has been shown to be present and integrated in the cellular genome, suggesting a causative role in the cancer development. Notably, MCPyV DNA has not been demonstrated in non-UV [30] or UV-light exposed melanomas [31], [32], [33], [34], in prostate cancer [35], in lymphoid tissues and tumours [36], in neuroendocrine tumours of the skin, in pulmonary neuroendocrine carcinomas [37], or in a diversity of human tumours including 21 neuroblastomas [24], [38], all from small children (Xavier Sastre-Garau personal communication). Hence, so far none of these studies suggests an aetiological role of MCPyV in any of these tumours. Nevertheless, to our knowledge, no study has been conducted regarding the presence of MCPyV in tumours of the central nervous system.

It is not unlikely that a virus, and particularly a polyomavirus is a causative agent of a tumour that arises early in life [39], [40]. Furthermore, very recently, a report was published describing the presence of WUPyV and KIPyV DNA in CNS tissue from HIV positive, but not from HIV negative, individuals [41]. Nonetheless, in a separate study, MCPyV DNA was not found in the cerebrospinal fluid from HIV positive men [42].

The data reviewed above compelled us to investigate for the presence of KI, WU and MC polyomaviruses in childhood tumours more specifically CNS tumours and neuroblastomas.

CNS tumours are the most common solid tumours in children and account for 27% of all childhood malignancies, second only to leukaemias [43], [44]. The predominant types of CNS tumours in children are astrocytomas, medulloblastomas/PNET, brainstem gliomas and ependymomas [44]. Neuroblastoma represents 5.5% of all childhood malignancies and is one of the most common extra cranial solid tumours in childhood [43]. The majority of these tumours are of embryonal origin and affects children at a very young age, with a median age at 18 months, being the most common malignancy in infants [45]. Neuroblastomas are derived from the neural crest cells and develops in tissues along the sympathetic nervous system, most frequently in the adrenal glands, or elsewhere in the abdomen, chest, neck and pelvis [46].

Here, in a pilot study we report the first analysis for presence of KI, WU and MCPyV DNA in tumour samples of 25 consecutive patients diagnosed during 2008–2009 with CNS tumours and 30 patients with neuroblastomas diagnosed between 1991 and 2008 at the Karolinska University Hospital. In addition, sera from 18/25 patients with CNS tumours were analysed for antibody titres against MCPyV.

## Materials and Methods

### Ethic Statements

For all patients in this study, written informed consent was obtained. This study was approved by the Regional Ethical Committee of Stockholm, Sweden.

### Patients, Tumour Material and Sera

Twenty-five fresh frozen CNS tumours from patients aged 0 to 18 years old were collected prospectively at the Astrid Lindgrens Children's Hospital within the Karolinska University Hospital between March 2008 and September 2009. In the operation room a biopsy measuring in average 2×2 mm could be collected from each tumour (a larger material was sometimes available). Additionally, in 14 cases, pieces from the ultrasound aspirate could be obtained at the end of the operation. For 18/25 patients blood samples could be collected at the operation room, or within 1 week after operation and stored −20°C. Patients and CNS tumours characteristics are detailed in Table 1 and grouped according to the Birch and Marsden classification scheme [47] in Table 2. In addition, 31 fresh frozen neuroblastomas from 30 patients, 0 to 11.5 years old, were collected prospectively between 1991 and 2008. More specifically, one patient had two biopsies from separate manifestations of multifocal disease [48]. The sample size of the neuroblastomas was considerably larger and allowed us to extract sufficient amount of DNA from each tumour.

**Table 1:** Characteristics of patients and their CNS tumours.

Patient	Gender/Age^1^	Localisation	Other diagnosis	Volume High×Length×Width (mm)	Histopathology (WHO)
1	F/5	Chiasma opticum		39×28×26	Pilomyxoid astrocytoma grade II
2	F/3	Intramedullary (C6-T9)		90×15×17	Ganglioglioma grade I
3	M/4/18*	4^th^ Ventricle		22×20×26 2	Ependymoma grade III
4	M/8/11	Front of 3^rd^ Ventricle, next to foramen Monroi	Neurofibromatosis type I, malignant optic nerve glioma	15×20×14	Pilocytic astrocytoma grade I
5	F/10	Bilateral subependymal, next to foramen Monroi	Tuberous sclerosis	28×28×45 2	Subependymal giant cell astrocytoma grade I
6	M/1	Posterior fossa		50×40×56	Teratoid/rhabdoid tumour grade IV
7	F/4/13	Intramedullary (L2-L3)	Neurofibromatosis type II, meningioma, Bilateral acoustic neuroma	30×15×19	Schwannoma grade I (MIB index 10–12%)
8	M/1	Subependymal, next to foramen Monroi	Tuberous sclerosis	17×20×14	Giant cell strocytoma grade I
9	F/9	Left frontal, from chiasma to corner of side ventricle		39×28×37	Atypic neurocytoma grade II
10	M/6	4^th^ Ventricle		29×23×26	Metastatic medulloblastoma grade IV, classic variant
11	M/5	Cerebellum		63×57×53	Pilocytic astrocytoma grade I
12	M/2/11	Back side of left frontal lobe and medullar	Leptomeningeal oligodendromatosis	15×10×16	Oligodendroglioma
13	M/6	Posterior fossa		23×19×18	Ependymoma grade III
14	M/0/2	Left subependymal, next to foramen Monroi	Tuberous sclerosis	9×14×12	Subependymal giant cell astrocytoma grade I
15	M/5	Pons		32×32×30	Pilocytic ponsglioma grade I
16	M/4	Frontoparietal right		26×27×22	Dysendrioblastic neuroepithelial tumour
17	F/4/8*	3^rd^ Ventricle and hypothalamus		31×33×21	Pilocytic astrocytoma grade I
18	M/3	Posterior fossa		30×35×40	Metastatic medulloblastoma grade IV, classic variant
19	M/8/11*	Posterior fossa		30×37×31	Pilocytic astrocytoma grade I
20	M/15	Left frontal supratentorial		57×52×85	Anaplastic oligoastrocytoma with undifferenciated component grade III-IV or small cell glioblastoma with undifferenciated component grade IV
21	M/14	Posterior fossa and 4^th^ ventricle		29×19×21	Metastatic Medulloblastoma grade IV, classic variant
22	F/2	Left frontal lobe		52×49×41	Supratentorial primitive neuroectodermal tumour (PNET) grade IV
23	M/5/12*	Intramedullary (C0-C6)		50×9×12	Cervicospinal astrocytoma grade I
24	M/3	Intramedullary (T5-T7)		50×70×50	Ependymoma grade II
25	M/0	Frontal bilateral		45×60×80	Desmoplastic Infantil tumour

1 Age at diagnosis/age at operation (* relapsing tumour).

2 Multiple nodules.

**Table 2:** Relative frequencies of tumour biopsies in the main diagnostic groups for CNS tumours as compared to their frequencies in the whole of Sweden.

CNS tumours Diagnostic groups*	Karolinska University Hospital 2008–2009	VCTB 1984–2005**
Ependymoma	3 (12%)	10.5%
Astrocytoma	11§ ^1^ (44%)	44.6%
Medulloblastoma/PNET	5 (20%)	18.8%
Other Gliomas	2§ ^2^ (8%)	10%
Others specified neoplasms	3 (12%)	13.5%
Unspecified intracranial/intraspinal neopl.	1§ ^3^ (4%)	2.5%
Total	25 (100%)	

*according to the “classification scheme for childhood cancer” [47].

**from the Swedish Childhood CNS Tumour Working Group (VCTB) [44].

§ Patients with tumours that can be due to inheritable diseases.

1 3/11 patients had Giant cell astrocytoma with Tuberous Sclerosis, and 1/4 had Pilocytic astrocytoma with Neurofibromatosis type I.

2 1/2 patients had Leptomeningeal oligodendromatosis.

3 this patient had Lumbar Schwannoma with Neurofibromatosis type II.

### DNA Extraction from Fresh Frozen Samples

The QIAamp DNA Mini Kit 50 (QIAgen®) (QIAGEN AB, Solna, Sweden) for blood and tissues was used according to the manufacturer's protocol to perform the extraction.

### Verification of Amplifiable DNA

To avoid false negative results due to DNA unsuitable for PCR analysis, a control PCR with human ribosomal gene S14 primers [49], [50] was run with 100–120 ng DNA for all samples.

### PCR Assay for the Detection of KIPyV and WUPyV

To detect KIPyV or WUPyV simultaneously, a standard PCR described previously [30] was used with a single primer pair hybridizing to a conserved genomic region shared by KIPyV and WUPyV in the gene encoding the capsid protein VP1, KIPyV2263F (5′-TTGGATGAAAATGGCATTGG-3′) and KIPyV2404R (5′-TAACCCTTCTTTGTCTAAAATGTAGCC-3′). The KIPyV2404R primer has an A/G mismatch with published WUPyV sequences at base number 20 (underlined). The resulting amplicon was 142 bp. The reaction mix was added to 100 ng of template DNA. As a positive control, KIPyV bp 1493–2653 was cloned into pcDNA3 [30].

### PCR Assay for the Detection of MCPyV

To detect the presence of MCPyV DNA, a standard PCR described previously [30] was used with a single primer pair hybridizing the N-terminal part of the LT region of MCPyV, MCPyLT1709.F (5′-CAGGCATGCCTGTGAATTAGGATG-3′) and MCPyVLT1846.R (5′-TCAGGCATCTTATTCACTCC-3′). The plasmid, pUC57MC1 [30], was used as a positive control. The reaction mix was added to 100 ng of template DNA. The resulting amplicon was 138 bp.

### Real-Time PCR Assay for the Detection of MCPyV

A hydrolysis probe–based, real-time PCR (rtPCR) designed to target the large T antigen (LT) gene, described previously, was used [26]. Reactions were performed with 100 ng of template DNA.

### MCPyV Serology Measured by a Neutralization Assay

A neutralization assay described previously [51] was used for detection of anti MCPyV antibodies. Titres were calculated using Prism software (GraphPad) from sigmoidal curves of four-fold serial dilutions of the sera.

## Results and Discussion

In this study, we have searched for a possible association of KI, WU or MC polyomaviruses with development of childhood tumours of the neural systems by investigating 25 CNS tumours and 31 neuroblastomas.

The presence of amplifiable DNA by a control PCR with human ribosomal gene S14 primers was confirmed in all tested samples, while DNA from KI, WU or MCPyV was not detected by regular PCR in any of the biopsies from the 25 CNS tumours, or from their corresponding 14 ultrasound aspirate specimens, or the 31 neuroblastomas (Table 3). Furthermore, for a better sensitivity, we complemented our standard MCPyV PCR with a real-time PCR for MCPyV. All our samples remained MCPyV DNA negative in this PCR with one exception: a sample with a Ct value of 37.8. It was derived from an ultrasound aspirate, while the corresponding biopsy directly taken from the tumour in the brain was negative in the same PCR. Thus, the presence of MCPyV in the tumour cells could not be confirmed. Consequently, we concluded that none of the tested CNS or neuroblastoma tumour samples scored positive for any of the three tested viruses.

**Table 3:** Overview of all tumours and results from the specific PCRs testing.

Specimens	Diagnosis/age	KIPyV^1^	WUPyV^1^	MCPyV^1,2^
31	**Neuroblastomas**	0	0	0
25	0–3 years old			
3	4–6 years old			
0	7–9 years old			
3	9–11 years old			
25	**CNS tumours**	0	0	0^1^/1^2^ *
9	0–3 years old			
10	4–6 years old			
3	7–9 years old			
1	9–11 years old			
2	11–18 years old			

1 Tested by regular PCR, or ^2^ by quantitative real time PCR.

*Ct value 37.8 in ultrasound aspirate but negative in corresponding tumour biopsy.

In addition, we tested sera available from 18/25 CNS tumour patients for MCPyV-specific antibody responses (Table 4). Of the tested sera 56% were completely negative in a highly sensitive neutralization assay, which very accurately excludes false negative results and has been described previously [51]. Of the MCPyV antibody-positive sera, all but one had antibody titres within two fold of the geometric mean titre (2700) calculated from an adult population (between 47 and 75 years old) tested earlier [51]. The remaining patient had an antibody titre of 98,740, which, while greater than the titres of other subjects in this study, was still below the geometric mean titre (160,000) reported for patients suffering from MCPyV-associated Merkel cell carcinomas [51].

**Table 4:** MCPyV Serological results from the 18/25 patients with CNS tumours.

Patients	Age (years)	CNS tumours Diagnosic group*	MCPyV Antibody Titre (EC50)
1	5	Astrocytoma	4,264
2	3	Other neoplasm	223
4	11	Astrocytoma	4,223
5	10	Astrocytoma	98,740
6	1	Medulloblastoma/PNET	100
7	13	Unspecified neoplasm	100
9	9	Other neoplasm	100
10	6	Medulloblastoma/PNET	5,410
11	5	Astrocytoma	100
13	6	Ependymoma	3,990
14	2	Astrocytoma	1,933
15	5	Astrocytoma	100
17	8	Astrocytoma	100
20	15	Astrocytoma	100
21	14	Medulloblastoma/PNET	775
22	2	Medulloblastoma/PNET	100
23	12	Astrocytoma	100
25	0	Other neoplasm	100

*according to the “classification scheme for childhood cancer” [47].

In all the PCR-based assays all samples contained 100 ng of DNA equal to the DNA content of >16 000 cells. The methods used for detection of KI, WU and MC polyomaviruses, were highly sensitive and should be adequate for the detection of low quantities of virus DNA reaching the detection level of 0.1 virus/copy per cell or less. The PCR used for the simultaneous detection of the related KI and WU polyomaviruses is known to detect as few as 10 copies of a KIPyV VP1 gene containing plasmid [30] corresponding to a detection limit of <0.001 viral DNA copies/cell genome. For detection of MCPyV we performed a previously established PCR assay using primers situated outside of the helicase domain and generating an amplicon of 138 bp [30]. With this assay, 1000 copies of a plasmid, pUC57MC1, containing the corresponding region of LT could be detected corresponding to a detection limit of <0.1 viral DNA copies/cell genome. To confirm that the detection limits of these two assays were unaffected by the presence of cellular DNA, up to 1000 ng of genomic DNA was mixed with plasmid DNA containing viral sequences without any reduction in the sensitivity (data not shown). In addition, we also used a previously established real time quantitative PCR for MCPyV [26], where a plasmid control with 2 copies/reaction was reproducibly positive, corresponding to <0.0002 copies/cell genome. Lastly, all tumour biopsies were fresh frozen and demonstrated amplifiable DNA in a PCR using human ribosomal gene S14 primers and resulting in 150 bp amplimers.

To our knowledge this is the first time the presence of the three newly discovered polyomavirus has been investigated in childhood CNS tumours and so far the results are negative, indicating that MCPyV, KI and WU polyomaviruses do not play a major, persistent role in oncogenesis of childhood CNS tumours. The data should however be taken with caution, since our analysis includes a limited number of CNS tumours of several diagnostic subsets, although the proportion of each tumour group was fairly similar to that of the Swedish registry (Table 2). Furthermore it should be noted that 6/25 (24%) patients had a genetic cancer syndrome known to play a role for CNS tumour development (Table 1), a proportion above the average population.

The absence of MCPyV in neuroblastomas here supports the similar negative finding in the 21 neuroblastomas of Sastre-Garau et al. [24] and suggests that MCPyV does not play an oncogenic role in neuroblastoma. Our report also complements with details of the age distribution of the neuroblastoma patients (Table 3) and that KI and WU polyomaviruses do not play an oncogenic role in neuroblastoma either.

In conclusion, in this pilot study MCPyV, KI and WU have so far not been shown to be causative of childhood CNS tumours or neuroblastomas. However, additional data are warranted and we do not exclude that other still not yet detected polyomaviruses could be present in these tumour types.
